# Association of regional cerebral oxygen saturation and postoperative pulmonary complications in pediatric patients undergoing one-lung ventilation: A propensity score matched analysis of a prospective cohort study

**DOI:** 10.3389/fped.2022.1077578

**Published:** 2022-12-08

**Authors:** Shanshan Li, Jianmin Zhang, Jing Hu, Lijing Li, Guoliang Liu, Tiehua Zheng, Fang Wang, Lin Liu, Gan Li

**Affiliations:** Department of Anesthesiology, Beijing Children's Hospital, Capital Medical University, National Center for Children's Health, Beijing, China

**Keywords:** pediatric patients, regional cerebral oxygen saturation, postoperative pulmonary complications, airway management, one-lung ventilation (OLV)

## Abstract

**Background:**

Previous studies of the relationship of regional cerebral oxygen saturation (rScO2) and postoperative pulmonary complications (PPCs) in pediatric patients are not well established, and further investigation is warranted. The aim of this prospective study was to determine whether a decrease in intraoperative rScO2 is associated with PPCs in children undergoing thoracoscopic surgery requiring one-lung ventilation (OLV).

**Methods:**

One hundred and six children of ages 3 months to 8 years who received one-lung ventilation were enrolled in the study. Upon entering the operating room, regional cerebral oxygen saturation was continuously monitored bilaterally by near-infrared spectroscopy. Patients were divided into low rScO2 (L-rScO2) or high rScO2 (H- rScO2) groups according to whether the lowest intraoperative rScO2 value was 15% lower than the baseline value. Outcome is defined as PPCs occurring within 7 days after surgery.

**Results:**

After propensity score matching, 23 pediatric patients with decreased rScO2 and 46 pediatric patients without a decrease in rScO2 were included in this study. According to logistic regression analysis, patients in the H- rScO2 group were less likely to have PPCs than those in the L-rScO2 group (OR = 3.16; 95% CI = 1.05–9.5; *P* = 0.04). Moreover, intraoperative rScO2 reduction was associated with an increase in the severity of PPCs (OR = 3.90; 95% CI = 1.19–12.80; *P* = 0.025).

**Conclusions:**

The decrease in regional cerebral oxygen saturation during surgery increases the likelihood of postoperative pulmonary complications.

## Introduction

The growth in popularity of video-assisted thoracic surgery (VATS) in pediatrics has increased the need for OLV. Although CO2 is usually infused into the chest during VATS to form a CO2 pneumothorax, optimal lung isolation improves surgical visualization and is a key factor in avoiding conversion from thoracoscopic to open-heart surgery. Due to the specificity of the surgical site and location, thoracoscopic surgery is more likely than other types of surgery to cause intraoperative fluctuations in oxygen saturation, leading to postoperative pulmonary complications (PPCs). The incidence of postoperative pulmonary complications in thoracic surgery is about 31.4% (1) and is one of the major causes of postoperative complications, prolonged hospital stays and mortality. Intraoperative hypoxemia is an independent risk factor for postoperative pulmonary complications (2). In addition, previous studies have shown that regional cerebral oxygen saturation is an earlier and more sensitive indicator of hypoxemia than pulse oximetry (SpO2) (3).

The potential effects of CO2 pneumothorax, including collapsed lung on the operative side, and reduced ventilation on the cardiopulmonary system of underdeveloped pediatric patients (especially neonates) are a matter of great concern when performing thoracoscopic surgery on pediatric patients. Monitoring rScO2 in pediatric patients in the operating room is becoming increasing common due to advances in intraoperative monitoring techniques. However, there are few studies on the impact of intraoperative rScO2 on postoperative clinical outcomes in the pediatric population.

Studies monitoring cerebral oxygen saturation during one-lung ventilation have focused on adults, and studies on postoperative complications have focused on postoperative cognitive impairment. There are few reports on the incidence, risk factors, and regression of pulmonary complications after one-lung ventilation thoracoscopic surgery in infants and children. There are significant anatomical, physiologic, psychological and pharmacological differences in children and adults; and the younger the age, the more pronounced the differences. Further research and application in monitoring rScO2 and PPC in pediatric patients is warranted. The aim of this study was to assess the association of decreased rScO2 and PPC events in children 8 years old and younger.

## Materials and methods

A total of 106 patients who received one-lung ventilation under general anesthesia between February 2021 and December 2021 were enrolled in this study. These patients were 3 months to 8 years of age, had American Society of Anesthesiologists physical status I to III, and underwent thoracoscopic mediastinal mass resection or lobectomy. Exclusion criteria were any of the following: (i) respiratory infection in the last two weeks, (ii) severe preoperative pulmonary disease, (iii) congenital cardiopulmonary disease, history of asthma, etc. (iv) preoperative clinically significant abnormalities in coagulation, liver and kidney function, (v) preoperative SpO2 below 95% while under aspirated air, and (vi) intraoperative conversion to open-heart surgery. Patients were divided into H-rScO2 and L-rScO2 groups according to whether intraoperative rScO2 decreased by 15% from baseline for 60 consecutive seconds. Baseline rScO2 was defined as the median value of the data recorded within 5 min prior to skin incision, when the anesthetized child was undergoing mechanical ventilation with one-lung ventilation.

All children were anesthetized and mechanically ventilated in the operating theatre and received standard monitoring. Anesthesia was induced with atropine (0.01 mg/kg), propofol (3 mg/kg), sufentanil (0.3 *μ*g/kg) and cisatracurium (0.1 mg/kg). Invasive arterial monitoring and end-expiratory carbon dioxide monitoring were performed. During the procedure, propofol was used to control the depth of anesthesia and remifentanil was used for intraoperative analgesia. Anesthesia was maintained with propofol (8–10 mg kg^−1^min^−1^) and remifentanil (0.2–0.4 *μ*g kg^−1^min^−1^). ABP was maintained within 15% of baseline levels. If the heart rate decreased more than 15%, atropine 0.01–0.02 mg/kg was given. The 5F Arndt endobronchial blocker was used for one-lung ventilation. The bronchial blocker was placed outside the tracheal tube and then guided into the bronchus on the surgical side using a fiberoptic bronchoscope. After lateral recumbency, one-lung ventilation was initiated with a tidal volume of 6–8 ml/kg, an increase of 20%–30% in respiratory rate without PEEP, and the inspired oxygen concentration was adjusted to 100%. Mechanical ventilation was maintained in volume-controlled mode in all patients, with constant flow during inspiration and no pressure regulation. After completion of the thoracoscopic procedure, both lungs were ventilated by evacuating the air from the Arndt blocker balloon. Postoperatively, the lungs were fully inflated and chest drains were left in place to ensure unobstructed drainage. In terms of fluid management, both groups were treated identically. To avoid over-infusion, only crystalloid fluids were used intraoperatively; no colloid fluids or blood products were used. At the time the patient entered the operating room (T1), the rScO2 was recorded. rScO2 was also recorded after induction of anesthesia intubation (T2), after lateral recumbency (T3), after one-lung ventilation (T4), 5 min after the start of surgery (T5), 15 min after the start of surgery (T6), 30 min after the start of surgery (T7), and at the end of one-lung ventilation (T8). The lowest value of rScO2 during surgery was recorded. Postoperatively, patients were admitted to the PACU for extubation. All patients received intravenous analgesia and antiemetic treatment with sufentanil (0.04 *μ*g kg^−1^min^−1^) and tropisetron (4 *μ*g kg^−1^min^−1^) for 48h postoperatively.

The medium sensor of the FORE-SIGHT® MC-203°C NIR monitor (NIRS, CASMED, United States) was placed 1 cm above the bilateral brow arches and adjusted as necessary to continuously monitor rScO2 on the left and right sides. Bilateral cerebral oxygen saturation was continuously monitored as the patient entered the operating room, and the data was finally analyzed using the average of the rScO2 values. As the rScO2 data was used only for research purposes, the surgical, anesthetic, and nursing staff were unaware of the real-time monitoring results. All patients were evaluated for heart rate (HR), arterial blood pressure (ABP), pulse oxygen saturation (SpO2), and end-tidal carbon dioxide pressure (PetCO2). In addition, postoperative laboratory data, imaging data, and vital signs were collected until the patient was discharged.

The primary outcome of this study was the incidence of PPCs within seven days postoperatively, and PPCs were graded on a scale of 0 to 4 (4). Grade 0 indicated that no PPCs occurred. Grades 1–4 represented PPCs of varying severity ranging from mild to severe. Grade 1 included a dry cough, microatelectasis, and dyspnea; grade 2 included a productive cough, bronchospasm, hypoxemia, atelectasis, and hypercarbia; grade 3 included pleural effusion, pneumonia pneumothorax, and postoperative reintubation, and grade 4 showed ventilatory failure (Table A). Complications were evaluated in detail using chest radiographs or CT (if available), laboratory data, clinical signs, and vital signs.

Sample size estimates were based on preliminary results from the first 33 pediatric patients. The ratio of children with and without a decrease of rScO2 was approximately 1:2.3, and the incidence of PPCs in children with or without a decline in rScO2 was about 40% and 15% respectively. Therefore, a total of 101 patients were required to detect a 25% difference with a power of 80% using a two-side proportion test at an alpha level of 0.05. Allowing for a 5% rate of attrition, the total sample size was increased to 106 patients.

All data were analyzed using SPSS version 24.0. Continuous variables that follow a normal distribution are expressed as mean and standard deviation. They are otherwise described as the median and interquartile range (IQR). The categorical variables are presented as frequency and percentage. A *t*-test or Mann-Whitney *U* test was used to determine whether the two sets of quantitative data differed according to whether the data conformed to a normal distribution. The *χ*^2^ test was used to test categorical data significance, and a Fisher's exact test was used when the sample size was *n* < 40 or the theoretical frequency was T < 1. Ordered multi-categorical logistic regression was used to analyze the relationship between the severity of PPCs and rScO2. Effects of the data were quantified by odds ratios (OR) and 95% confidence intervals (CI). *P* < 0.05 was considered to be a statistically significant difference.

To further confirm the association of regional cerebral oxygen saturation and postoperative pulmonary complications, we conducted a sensitivity analysis using propensity score matching (PSM) as the H/L groups differed significantly across baseline characteristics. We established a multiple regression model which included 9 baseline variables including age, sex, body weight, type of operation (lobectomy or resection of a mediastinal mass), total infused fluid, estimated blood loss, urine output, total operation time, and anesthesia time. Patients from the L-group were matched with patients from the H-group using one-to-two nearest neighbor matching with a caliper of 0.05.

## Results

**Figure 1:**
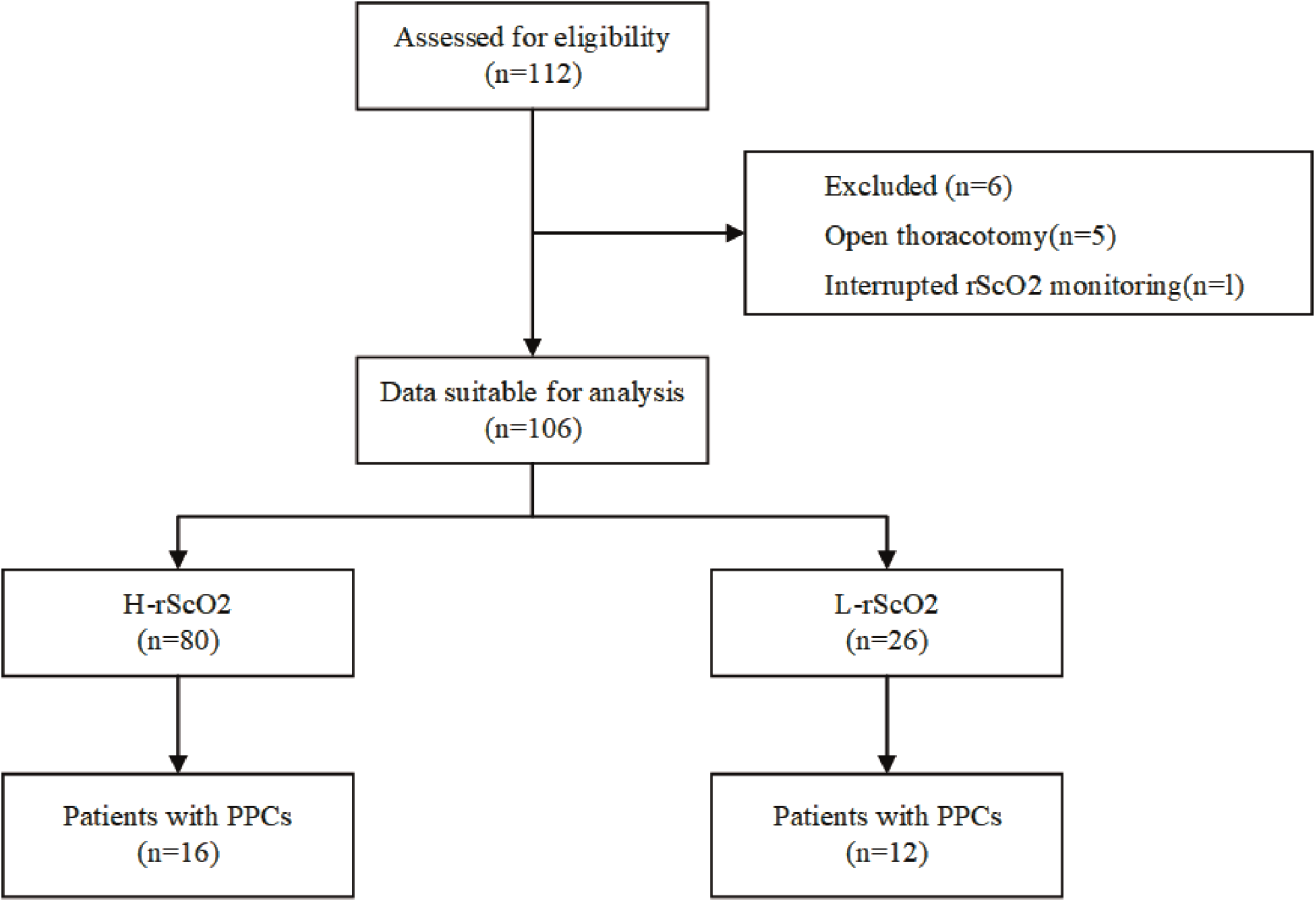
Flow diagram of the study participants.

Initially a total of 112 patients were included in this study. After excluding 5 patients for whom the surgical procedure was changed from thoracoscopic surgery to open-heart surgery, and 1 patient with missing data, 106 children were included in the final analysis (Figure 1). After propensity score matching, there were 23 children in the L-rScO2 group and 46 children in the H-rScO2 group. Comparing the baseline characteristics and intraoperative variables of all patients in the H-rScO2 and L-rScO2 groups, no significant differences were found in the general data of these two groups (Table 1).

**Table 1:** Baseline characteristics and intraoperative variables of all patients before and after propensity score matching. Data are presented as mean (standard deviation), median (inter-quartile range), or n (%). rScO2: regional cerebral oxygen saturation.

Variable	Before propensity score matching			After propensity score matching (1:2)		
	L-rScO2 group (*n* = 26)	H-rScO2 group (*n* = 80)	Standardized difference	L-rScO2 group (*n* = 23)	H-rScO2 group (*n* = 46)	Standardized difference
Age (years)	3 [0.87–3.75]	4 [1–5]	0.380	3 [1–4]	3 [0.84–4]	0.210
Sex (male)	11 (42.31%)	46 (57.5%)	0.307	10 (43.48%)	24 (52.17%)	0.175
Body weight (kg)	13.75 [9.62–15.38]	15.25 [12–21.12]	0.324	14 [10.5–15.25]	13.25 [10.25–17]	0.106
Type of operation		0.102			<0.001	
Lobectomy	14 (53.85%)	39 (48.75%)		11 (47.83%)	22 (47.83%)	
Mediastinal mass resection	12 (46.12%)	41 (51.25%)		12 (52.17%)	24 (52.17%)	
Total infused fluid (ml)	200 [150–300]	250 [150–412.5]	0.918	200 [150–3250]	200 [150–300]	0.277
Estimated blood loss (ml)	2 [2–2]	2 [2–2]	0.619	2 [2–2]	2 [2–2]	0.299
Urine output (ml)	75 [12.5–137.5]	50 [20–100]	0.970	100 [15–125]	50 [16.25–100]	0.287
Total operation time (min)	80.5 [60.5–103.75]	82.5 [52.25–125]	0.041	75 [60–100]	75 [47–111.25]	0.026
Anesthesia time (min)	120 [97–139.25]	120 [80–160]	0.015	120 [93–136]	120 [76.25–145]	0.022

A total of 28 (26.5%) children developed pulmonary complications within 7 days following surgery. After propensity score matching, 9 of these children were in the H-rScO2 group and 10 were in the L-rScO2 group. All pneumothoraxes and pleural effusions occurred on the same side as the operation. According to the results of binary logistic regression analysis, patients in the L-rScO2 group were significantly more likely to have PPCs than those in the H-rScO2 group (OR = 3.16; 95% CI = 1.05–9.5; *P* = 0.04). Intraoperative reduction of rScO2 was associated with an increase in the severity of PPCs according to the results of ordered multicategorical logistic regression analysis (OR = 3.90; 95% CI = 1.19–12.80; *P* = 0.025) (Table 2).

**Table 2:** Comparisons of PPCs between the H-rScO2 and L-rScO2 groups after propensity score matching.

Outcome	L-rScO2 group (*n* = 26)	H-rScO2 group (*n* = 46)	Odds ratio (95% CI)	*P*-value
Level of PPCs	10 (43.48%)	9 (19.57%)	3.90 (1.19–12.80)	0.025
Grade 1	6 (26.09%)	5 (10.87%)	4.36 (0.13–150.75)	0.415
Grade 2	3 (13.04%)	3 (6.52%)	15.84 (0.43–590.06)	0.134
Grade 3	1 (4.35%)	1 (2.17%)	100.50 (1.94–5204.62)	0.022
Grade 4	0 (0.0%)	0 (0.0%)	–	–

**Figure 2:**
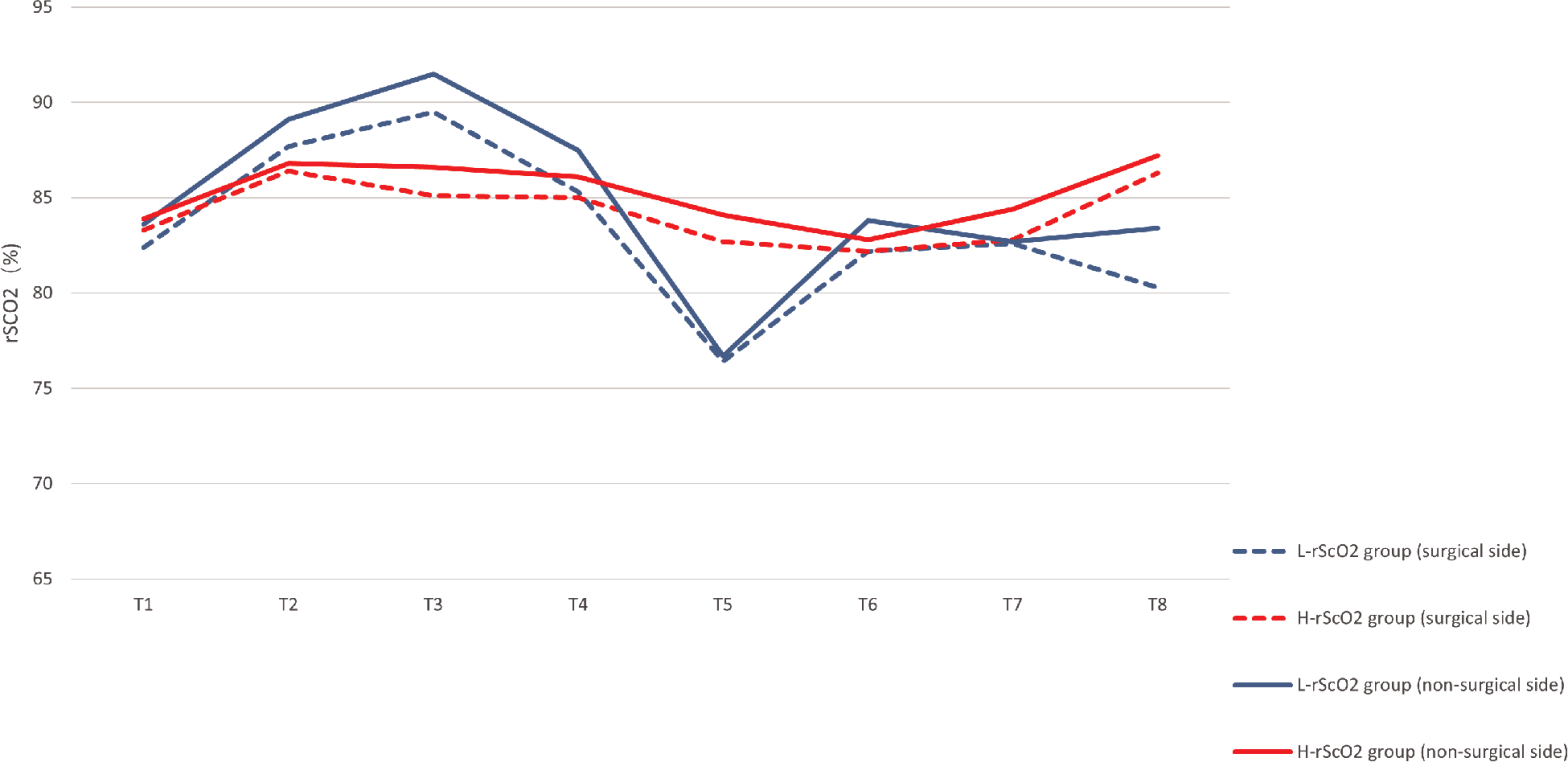
Comparison of intraoperative rSCO2 at each time point.

There were significant differences in rScO2 at each time point of the study: rScO2 increased after induction and intubation (T2). However, rScO2 did not fluctuate significantly in the lateral position (T3), while rScO2 decreased slightly at the start of one-lung ventilation (T4). rScO2 decreased significantly 5 min into the procedure (T5), and rScO2 increased slowly thereafter as the procedure progressed (Figure 2). By analyzing the time of onset of the intraoperative nadir for rScO2 in the L-rScO2 group, we found two nadirs of intraoperative rScO2 in the L-rScO2 group (7.7%) which occurred after one-lung ventilation. 21 of these (80.8%) occurred within 5 min of the start of surgery, and three (11.5%) occurred during intraoperative maneuvers. According to analysis with the Mann-Whitney *U* test, there were no significant differences in heart rate, blood pressure, PETCO2, or oxygen saturation which were routinely monitored at each time point for the two groups (Table 3).

**Table 3:** Intraoperative monitoring data. MAP: mean arterial pressure. HR: heart rate. PetCO2: end-tidal carbon dioxide pressure. SpO2: pulse oxygen saturation.

	Parameters	L-rScO2 group (*n* = 26)	H-rScO2 group (*n* = 80)	*P*-value
T1	MAP (mmHg)	–	–	–
	HR (bpm)	113.5 [108.25–135]	108 [97–120.75]	0.069
	PetCO2 (mmHg)	–	–	–
	SpO2 (%)	100 [100–100]	100 [100–100]	0.418
T2	MAP (mmHg)	64 [60–67]	65.5 [60–71]	0.591
	HR (bpm)	117.5 [107–143.25]	111.5 [101–131]	0.15
	PetCO2 (mmHg)	41 [38–44]	38 [35.75–42]	0.231
	SpO2 (%)	100 [100–100]	100 [100–100]	0.72
T3	MAP (mmHg)	66 [60.75–71]	65 [61–72]	0.944
	HR (bpm)	117.5 [101.5–138.75]	110.5 [94–134.25]	0.201
	PetCO2 (mmHg)	43 [38.5–44.75]	40 [36.75–41.25]	0.006
	SpO2 (%)	100 [100–100]	100 [100–100]	0.92
T4	MAP (mmHg)	63.5 [57.25–68.75]	65 [60–74]	0.187
	HR (bpm)	120 [102.25–137.25]	110 [100.75–138.5]	0.544
	PetCO2 (mmHg)	42 [39.25–44]	41 [35–46.25]	0.614
	SpO2 (%)	100 [100–100]	100 [100–100]	0.729
T5	MAP (mmHg)	60.5 [55.25–71.5]	68.5 [61–77.5]	0.032
	HR (bpm)	124.5 [105–144.75]	120.5 [111–140.5]	0.769
	PetCO2 (mmHg)	41 [35.25–44.75]	44 [37–46.25]	0.203
	SpO2 (%)	100 [99–100]	100 [99–100]	0.333
T6	MAP (mmHg)	65 [56.25–73.25]	69 [60–77]	0.126
	HR (bpm)	122.5 [109.25–139.5]	118 [108.75–141.25]	0.959
	PetCO2 (mmHg)	43.5 [38–50]	47 [42–52.25]	0.231
	SpO2 (%)	100 [99–100]	100 [100–100]	0.312
T7	MAP (mmHg)	64.5 [56–69.5]	63 [58–71.25]	0.421
	HR (bpm)	120 [112.5–139.5]	124 [106–136.25]	0.643
	PetCO2 (mmHg)	43 [39–50]	44 [40–52]	0.482
	SpO2 (%)	100 [99–100]	100 [100–100]	0.194
T8	MAP (mmHg)	75 [64.5–79.5]	72 [65–83]	0.376
	HR (bpm)	112 [100.5–126.5]	104.5 [91–123.5]	0.232
	PetCO2 (mmHg)	39 [34.75–47]	38 [35.75–43]	0.387
	SpO2 (%)	100 [100–100]	100 [100–100]	0.112

## Discussion

This prospective cohort study showed that decreases in intraoperative rScO2 are associated with an increased incidence of PPCs in children undergoing thoracoscopic procedures requiring OLV.

Intraoperative hypobaric oxygen saturation is associated with one-lung ventilation. During thoracoscopic surgery, patients are usually ventilated with one lung in the lateral position, which results in a significant ventilation/flow ratio imbalance that may lead to hypoxemia, ventilation-perfusion imbalance, and ischemia-reperfusion of the lungs. At the same time, injection of CO2 into the thorax at the surgical site reduces negative intrathoracic pressure, or changes negative intrathoracic pressure to positive pressure. One-lung ventilation on the non-surgical side changes negative intrathoracic pressure to positive pressure, which leads to obstruction of venous return to the head and neck with consequent increases in intracranial venous pressure and compromised cerebral perfusion (5). In addition, rScO2 reflects the balance of supply and demand in brain metabolism, and children are prone to fluctuations due to higher brain oxygen consumption and imperfect cerebral blood flow autoregulation mechanisms (6). These factors may contribute to decreases in rScO2. The intraoperative oxygen saturation of the children in this study was mostly 100%, which may be due to better preoperative lung function, the relatively short duration of surgery, and the inhalation of pure oxygen during one-lung ventilation. Intraoperative hyperoxia may lead to pulmonary atelectasis (7), but it has also been shown that inhalation of different concentrations of oxygen does not have an effect on the development of severe pulmonary atelectasis in mechanically ventilated children (8). In addition, all children underwent manual pulmonary resuscitation before the end of the procedure.

The physiology of the respiratory system in children differs from that of adults. Young children and infants are more prone to hypoxia during general anesthesia than adults. This is due to the fact their functional residual air volume is close to the residual air volume, and they are less tolerant of hypoxia. Moreover, the healthy airway in these children is more likely to close during inspiration. In addition, infants have a small thorax, a more compliant chest wall, and a small hydrostatic pressure difference between the thorax and abdomen in the lateral position, which affects diaphragmatic assistance (9). In addition, increased intraoperative oxygen consumption and technical issues related to pediatric lung isolation may lead to an increased risk of PPCs (10). As a result, the incidence of postoperative pulmonary complications is also higher in pediatric patients than in adults (11). It has been shown that the use of a tidal volume of 8 ml/kg and PEEP of 6 cmH2O during one-lung ventilation thoracoscopic surgery in children reduces the incidence of PPCs (12). However, a small tidal volume ventilation with a PEEP value which is inappropriate because it is set too high or too low, may increase the risk of pulmonary atelectasis and hypoxemia.

The PEEP setting has the potential to increase the risk of pulmonary atelectasis and hypoxemia (13). A standard strategy for protective lung ventilation in children has not been fully developed, and protective lung ventilation was therefore not used in this study.

The factors which affect cerebral oxygen saturation are age, hemoglobin concentration, oxygen saturation, end-expiratory carbon dioxide, body temperature, probe placement, and intraoperative position. In this study, we selected children of ages 3 months to 8 years, all of whom were placed in the lateral position intraoperatively, and probe positions were all located 1 cm above the bilateral brow arches. On this basis, it was found that conventional intraoperative monitoring such as heart rate, blood pressure, and oxygen saturation did not change simultaneously when cerebral oxygen saturation was reduced by more than 15%, which did not reflect cerebral hypoxia in a direct and real-time manner. This result is consistent with previous studies (14, 15). As the partial pressure of carbon dioxide increases, the plateau phase of the cerebrovascular regulation curve rises (16). That is, as the partial pressure of carbon dioxide rises, cerebral blood flow increases at the same perfusion pressure. In addition, some studies have shown that intraoperative maximum partial pressure of end-expiratory carbon dioxide >50 mmHg is an independent risk factor for pulmonary complications after one-lung ventilation in infants and children (17). However, in this study, there was no significant difference in PetCO2 in these two groups (*P* > 0.05) and PetCO2 was less than 50 mmHg. We therefore concluded that intraoperative PetCO2 in this study did not affect the study results. It may be that children have relatively better lung compliance and better tolerate a range of internal volume injuries. In the present study, rScO2 did not decrease significantly in most children after one-lung ventilation, probably because of the short time interval between one-lung ventilation (T4) and the start of surgery at 5 min (T5). It takes some time for the lung to collapse on the operated side, and for a series of physiologic changes to occur. Moreover, the time required for lung atrophy on the operative side and the subsequent series of physiologic changes, resulted in a less significant decrease in rScO2 at T4. A significant decrease in rScO2 in most children occurred within 5 min of the start of surgery, and this may be due to the fact that on the one hand, the lung on the operated side is well atrophied at this time, and the ventilation/blood flow ratio is out of balance, leading to intrapulmonary shunting and resulting in hypoxia. On the other hand, the surgeon is injecting CO2 through the thoracoscopic incision and establishing an artificial CO2 pneumothorax to achieve a better surgical view.

There are only limited clinical data on the relationship of rScO2 and PPCs in children. One study showed that a decrease in cerebral oxygen saturation in adults receiving one-lung ventilation occurs routinely (18). However, in most cases, finger pulse oxygen saturation remains within clinically acceptable levels. Furthermore, it has been shown that a decrease in the rScO2 values is positively correlated with PPCs (19) and there is no correlation between regional cerebral oxygen saturation and cardiac output or other hemodynamic variables. However, that study included only adults and the sample size was a small. This study was thus not informative in regard to children.

The results of this study show that intraoperative hypoxia is associated with occurrence of PPCs. This association may be due to the intraoperative occurrence of pulmonary atelectasis and airway obstruction, with consequent occurrence of a reduction in functional residual air volume, while allowing a high level of lung instability during intraoperative ventilation (2). At the same time, the protective mechanisms of the brain (autoregulation) may make it the last organ to show impaired blood flow and/or oxygenation. This is because the hierarchical nature of blood flow distribution among organ systems ensures preferential perfusion of the brain at the expense of other organs (20, 21). Therefore, those patients who exhibit reduced oxygenation to the brain may have inadequate antecedent perfusion to other organs, which may also contribute to the development of PPCs. There is also the possibility that some children may have undiagnosed infections or other problems, which may lead to intraoperative hypoxia and eventually to the development of PPCs.

Earlier studies have shown intraoperative hypoxemia to be an independent risk factor for postoperative pulmonary complications (2). Recent studies have found a significant correlation between intraoperative rScO2 and SpO2. The decline in rScO2 occurs earlier, move rapidly and with greater sensitivity than the decline in SpO2 (3). In the present study, we observed some discordance in the changes in SpO2 and rScO2. Pulse oximetry is a routinely used monitor of blood oxygen saturation. However, it does not accurately respond to human hypoxia and may trigger or exacerbate cerebral hypoxia in practice. Routine intraoperative monitoring of rScO2 provides time for anesthesiologists to promptly manage hypoxemia and prevent further exacerbation of hypoxia.

There are some limitations to this study. First, we used a 5 Fr Arndt blocker outside the tracheal tube for one-lung ventilation in all children, so the upper age of study participants was limited at 8 years. Although this reduces the number of variables and makes results easier to interpret, it raises uncertainty as to whether this outcome is appropriate for application with older pediatric patients. Secondly, because of the limited forehead area in infants and children, and considering the fact that intact nerve fiber myelin is not fully formed until 2 to 7 years of age in pediatric patients, adult data simply cannot be applied. We therefore did not routinely use the bispectral index (BIS) to monitor anesthetic depth, and this raised the possibility that some of the variability in operative heart rate blood pressure was due to insufficient anesthetic depth. Third, we focused only on PPCs and neglected other postoperative complications of thoracoscopic surgery, especially postoperative cognitive dysfunction.

In conclusion, this prospective cohort study shows that a decrease in rScO2 during OLV is associated with PPCs. Continuous intraoperative real-time monitoring of rScO2 may provide improvement in clinical management by providing information which will help reduce the actual duration of hypoxia in patients and to reduce the occurrence of PPCs. We recommend routine intraoperative rScO2 monitoring in children with risk factors for preoperative PPCs, and intervention should be undertaken before the rScO2 drops more than 15%, thus ensuring intraoperative oxygenation and improving patient prognosis.

## Data Availability

The original contributions presented in the study are included in the article/Supplementary Material, further inquiries can be directed to the corresponding author/s.
